# Toward multifunctional nanoplatforms based on layered double hydroxides (LDHs) for cancer therapy: From structural design to application

**DOI:** 10.1016/j.bioactmat.2025.08.034

**Published:** 2025-10-08

**Authors:** Nazila Biglari, Matineh Ghomi, Ehsan Nazarzadeh Zare, Elham Mahmoudi, Jianliang Shen, Pooyan Makvandi

**Affiliations:** a School of Biological Sciences, Universiti Sains Malaysia, 11800, Penang, Malaysia; b Department of Chemistry, Jundi-Shapur University of Technology, Dezful, Iran; c School of Chemistry, Damghan University, Damghan, 36716-45667, Iran; d The Quzhou Affiliated Hospital of Wenzhou Medical University, Quzhou People's Hospital, Quzhou, 324000, Zhejiang, China; e Center for Theoretical Physics, Khazar University, Baku, Azerbaijan; f National Engineering Research Center of Ophthalmology and Optometry, Eye Hospital, Wenzhou Medical University, Wenzhou, Zhejiang, 325027, China

**Keywords:** Nanoplatforms, Layered double hydroxides (LDH), Cancer therapy, LDH nanocarriers, Biocompatible nanomaterials, Targeted drug delivery

## Abstract

Layered double hydroxides (LDHs), known as anionic clays structured from metal hydroxide layers bearing positive charges and embedded anions are emerging as valuable tools in the field of cancer treatment. Owing to their inherent features such as extensive surface area, adjustable structure, ion-exchange potential, and compatibility with biological systems, these materials are well-suited for encapsulating and delivering various therapeutic agents including chemotherapeutic drugs, gene-based treatments, photosensitizers (PSs), and immune-modulating compounds in a controlled manner. This review offers an in-depth analysis of the latest developments in the design, preparation, and modification of LDHs for cancer-related applications with a special focus on their use in localized therapeutic delivery, gene transfer strategies, and multimodal therapeutic approaches. A novel dimension of LDH nanotechnology lies in its evolution from passive drug carriers to multifunctional theranostic systems capable of real-time imaging, environmental responsiveness, and multi-modal treatment delivery. Emerging innovations include synergistic photothermal-chemodynamic therapy, LDH-mediated immunotherapy, and CRISPR-Cas9 gene editing facilitated by the pH-sensitive and protective nature of LDHs. Additionally, hybridization with other nanomaterials and integration with AI-guided design and wearable biosensor technologies are opening new frontiers in personalized cancer care. Despite challenges such as *in vivo* stability and clinical translation, LDHs present transformative potential in revolutionizing precision oncology through safer, more effective, and individualized therapeutic strategies.

## Highlights

• Layered double hydroxides (LDHs) are versatile, tunable nanoplatforms for next-generation cancer therapeutics.
• LDH composition and surface engineering enable targeted drug delivery, gene editing, and theranostic applications.
• Advanced LDH systems combine photothermal, photodynamic, and CRISPR-Cas9 for synergistic cancer treatments.
• We critically assess the translational prospects and clinical pathways of LDH-based nanomedicines.

## Graphical abstract

**Figure:**
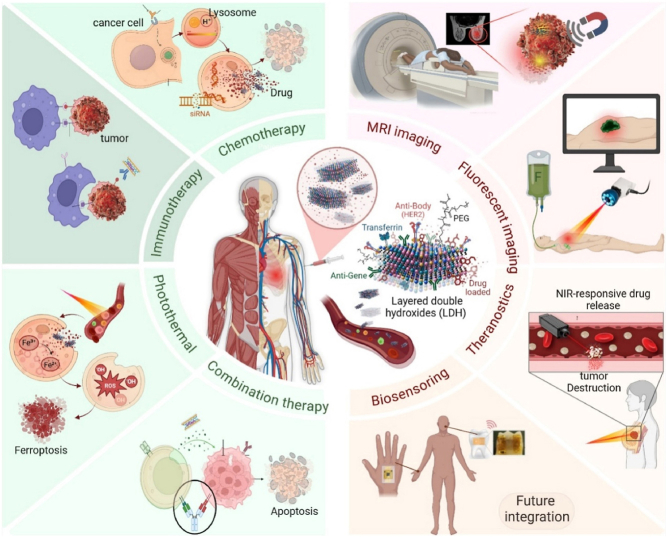

## 1 Introduction

Layered double hydroxides (LDHs) also known as hydrotalcite-like materials are a class of anionic clays composed of positively charged metal hydroxide layers and exchangeable interlayer anions [1,2]. Their two-dimensional structure, typically incorporating cations such as Mg^2+^, Al^3+^, or Fe^2+^, offers high surface area, tunable composition, and remarkable ion-exchange capacity [3,4]. These properties enable LDHs to host a broad range of anions including small molecule drugs, nucleic acids, and bioactive compounds within their interlayer spaces. The ability to encapsulate and protect sensitive therapeutics while enabling controlled, stimulus-responsive release has made LDHs an attractive platform in nanomedicine [[5], [6], [7], [8]].

Beyond single-agent therapy, LDHs can be engineered as multifunctional platforms for combination treatment, enabling the co-delivery of drugs with complementary mechanisms or integration with photothermal, photodynamic, or immune-based therapies [9,10]. Such versatility has stimulated growing interest in their application across the cancer treatment landscape [11,12]. Recent studies also demonstrate that LDHs can improve the pharmacokinetic profiles of loaded agents, enhance intracellular uptake, and minimize premature degradation which together contribute to improved therapeutic outcomes. Advances in synthesis and surface engineering continue to expand the design space for LDHs, allowing the creation of nanoplatforms with greater precision, adaptability, and clinical promise [13].

This review provides a comprehensive overview of recent advances in LDH-based nanoplatforms for cancer therapy. We explore the structural features that underpin their performance, synthesis and functionalization strategies for biomedical use, and mechanisms governing targeted and controlled drug release. Emphasis is placed on approaches that manipulate tumor-specific microenvironments and surface engineering to enhance therapeutic precision. Finally, we discuss the remaining challenges including stability, toxicity, and clinical translation, offering insights into how LDH nanoplatforms may reshape the future of cancer treatment.

## 2 Fabrication and functionalization of LDH nanoplatforms

LDHs are promising nanomaterials for biomedical use. Their stability, drug-loading, and bioavailability depend on how they are synthesized and modified. Understanding these processes is crucial for customizing LDHs for targeted drug delivery, gene therapy, and other applications [1,2]. Careful control of synthesis such as composition, size, and shape affects their performance. The right methods improve stability and efficiency, making LDHs more effective in clinical settings [14]. Functionalization, through chemical modifications or coatings, enhances biocompatibility and targeting, enabling controlled release and improved selectivity for diseased cells key for cancer therapy and regenerative medicine. By optimizing synthesis and functionalization, researchers can develop LDHs with better stability, targeted delivery, and therapeutic efficacy, supporting advanced healthcare innovations [1,2,15,16].

### 2.1 Synthesis of LDH nanoplatforms

Several synthesis techniques have been developed to engineer LDHs with tailored physicochemical characteristics suited for biomedical purposes. Each method offers distinct advantages in terms of composition control, particle size, crystallinity, and scalability.

***Co-precipitation*** is the most widely used and scalable method for synthesizing LDHs, offering fine control over chemical composition and crystallinity [17]. It involves dissolving metal salts such as magnesium, aluminum, or zinc nitrates in aqueous solution followed by the gradual addition of a pH-controlling base to induce hydroxide precipitation, forming layered structures stabilized by interlayer anions. Bioactive molecules or drug compounds can be incorporated *in situ* during this process by introducing them as intercalating anions or co-dissolving them in the reaction mixture, allowing simultaneous formation of LDH layers and intercalation of therapeutic agents. Adjusting metal cation ratios tunes critical physicochemical properties including charge density, crystallinity, and interlayer spacing which affect drug-loading efficiency. Reaction conditions such as temperature, stirring rate, and pH strongly influence particle size, morphology, and drug distribution within the LDH. Co-precipitation yields LDHs with reproducible and tunable compositions making it ideal for biomedical applications requiring consistency. However, the resulting LDHs often need further modifications to enhance dispersibility and bioavailability [18,19]. These can be addressed by optimizing synthesis parameters to produce uniform nanoscale particles and by post-synthesis functionalization with biocompatible polymers (e.g., PEG, chitosan) to improve colloidal stability and enable controlled drug release [17]. Overall, co-precipitation remains a highly versatile technique for generating reproducible LDH-based nanocarriers with efficient *in situ* drug incorporation, particularly valuable in cancer therapy [18].

***The hydrothermal method*** produces LDHs with enhanced crystallinity, uniform morphology, and superior temperature stability compared to those obtained by conventional co-precipitation [1]. In this approach, a precursor solution containing metal salts and a suitable base is sealed in an autoclave under controlled temperature and pressure, promoting the growth of highly ordered hydroxide layers [20]. Bioactive molecules or drug compounds can be incorporated either *in situ* by introducing the therapeutic agents directly into the precursor solution so that they are intercalated between the growing LDH layers during crystallization or post-synthetically by dispersing pre-formed LDHs in a drug-containing solution to enable ion-exchange or surface adsorption. The resulting well-ordered layered structures enhance drug loading efficiency and support sustained, pH-responsive release, crucial for biomedical applications. By adjusting synthesis variables such as processing time, temperature, and precursor ratios, LDHs can be tailored for specific structural and functional properties. While the hydrothermal method yields high-quality nanostructures with improved dispersibility, it requires specialized equipment and prolonged reaction times, limiting scalability for large-scale biomedical use [19,20]. Transitioning to continuous-flow hydrothermal synthesis and employing scalable precursors with in-line monitoring can address these challenges, enabling reproducible, cost-effective production without compromising material performance [20].

***Sol gel method*** provides a route for manufacturing LDHs with precise structural control. The technique entails dissolving metal-based precursors in a solvent followed by hydrolysis and condensation reactions that facilitate the development of a gel structure embedding LDH precursors. The gel is subjected to thermal treatment that promotes crystallization and the development of LDH nanosheets with controlled pore structure. This technique allows the incorporation of functional molecules such as organic ligands, polymers, or biomolecules directly into the LDH matrix during synthesis which enhances their biocompatibility and therapeutic efficacy [1]. The sol-gel technique enables the fabrication of nanomaterials characterized by exceptional purity and uniformity which are essential for biomedical applications that require precisely engineered nanocarriers. The ability to control the porosity and surface chemistry of LDHs synthesized *via* this method ensures that these materials exhibit improved ability to load drugs combined with customized release profiles. Nonetheless, optimizing the synthesis conditions in the sol-gel method is essential to avoid the aggregation or excessive growth of LDH nanosheets which can affect their performance in drug delivery and other biomedical applications [18]. These include using soft templates such as surfactants or polymers (e.g., PVP or PEG) to control particle size and dispersion, adjusting pH and precursor concentration to regulate nucleation and growth, and incorporating chelating agents to slow down metal ion hydrolysis. Additionally, conducting the process under mild temperature and stirring conditions can enhance uniformity and reduce agglomeration which lead to better control over LDH morphology and improved performance in drug delivery and biomedical applications [18,21].

***Microwave-assisted synthesis*** has emerged as a promising technique for the production of advanced nanocomposites with improved properties. This technique enables the simultaneous formation of LDH and reduction of graphene oxide, leading to the development of innovative nanohybrids. There are two main ways to carry out this synthesis. The first is the one-pot method, where hydrothermal crystallization of LDH occurs while reducing graphene oxide in a single process driven by microwave energy [22]. The second is the layer-by-layer technique which involves growing LDH directly onto graphene surfaces, resulting in composites with increased surface area and better electrochemical performance. Nanohybrids made of graphene and LDH through this microwave-assisted approach show significantly improved capacitance with specific values reaching up to 1055 F/g, notably higher than pure LDHs [23]. Additionally, LDH modified with ionic liquids demonstrates high polymerization rates, highlighting the versatility of this method for producing catalytic nanocomposites [24]. Overall, this synthesis technique offers many advantages such as faster production times and enhanced material properties. However, challenges like scaling up the process and maintaining uniformity in larger amounts need to be carefully considered when moving toward industrial applications.

**Fig. 1:**
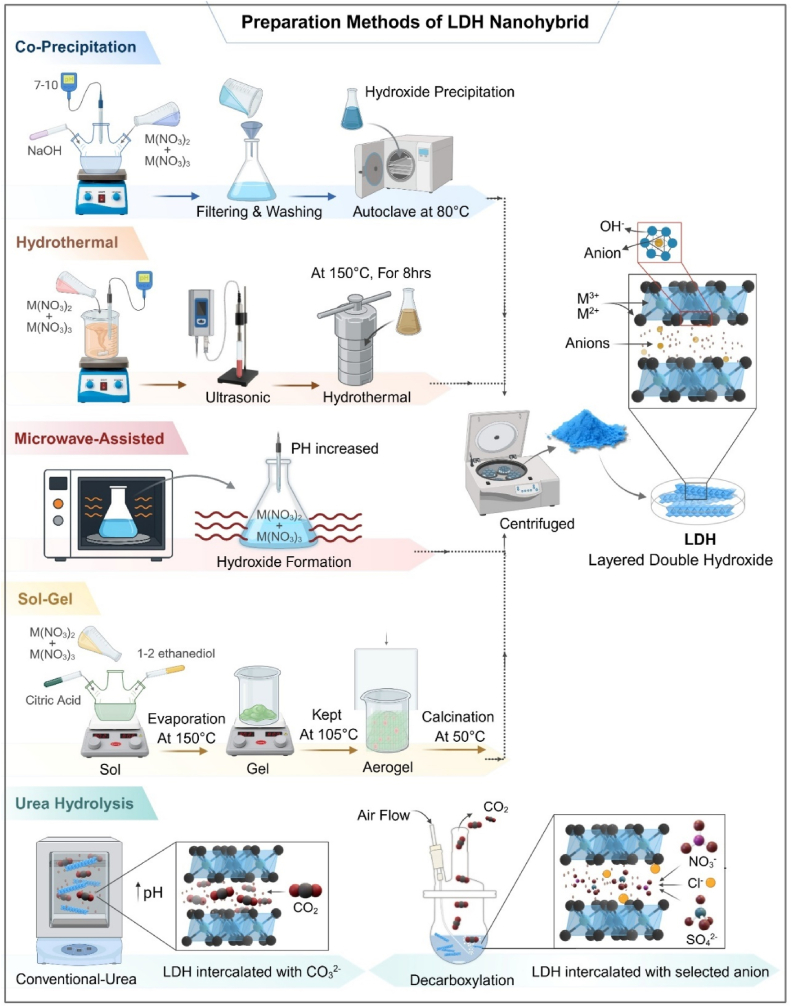
Schematic overview of various synthesis techniques employed for fabricating Layered Double Hydroxide (LDH) nanohybrids tailored for biomedical applications. Illustrated methods include co-precipitation, hydrothermal synthesis, microwave-assisted synthesis, sol–gel processing, and urea hydrolysis. Each method offers specific advantages in controlling the physicochemical properties such as crystallinity, particle size, and interlayer anion incorporation, which are critical for optimizing LDH performance in drug delivery and regenerative medicine [37].

***Urea hydrolysis*** acts as a smooth and uniform method for producing hydroxide and carbonate ions which help in forming layered double hydroxides (LDHs) when maintained at specific temperatures. The process involves a delicate balance between urea breakdown, metal ion interactions, and temperature control, all of which impact how the LDH crystals form and grow. This understanding connects the conditions used during synthesis to the final properties and performance of the LDHs, aiding in the design of improved catalysts for urea oxidation and energy-related uses [25]. The mentioned methods used to formulate LDH-based nanohybrids and outlined recent developments in their application are briefly shown in Table 1. Fig. 1 shows the synthesis method of LDH.

**Table 1:** Comparison of nanoparticle synthesis methods: scalability, particle size control, drug-loading efficiency, and clinical suitability.

Method	Scalability	Particle Size Control	Drug-Loading Efficiency	Clinical Suitability	Pros.	Cons.	Ref.
Co-precipitation	High	Moderate	High	Moderate	Simple and cost-effective- Good for large-scale production	Broad particle size distribution, Poor crystallinity in some cases	[[26], [27], [28]]
Hydrothermal	Moderate	High	High	High	Produces highly crystalline, uniform particles, Good drug loading	Requires high pressure/temperature, Less scalable	[29]
Sol-Gel	Low–Moderate	High	Moderate	Low–Moderate	Excellent control over morphology and porosity	Complex process, Limited scalability, High cost	[30]
Ion-Exchange	Moderate	High (post-synthesis)	Moderate–High	Moderate	Allows precise incorporation of sensitive bioactives	Multi-step process, Lower yield	[31,32]
Urea Hydrolysis	High	Moderate	Moderate	Moderate	Mild conditions, Suitable for heat, sensitive drugs	Longer reaction time, Moderate crystallinity	[33,34]
Microwave-assisted	Moderate	High	High	Under investigation	Rapid synthesis, Enhanced crystallinity	Equipment-dependent, Scale-up challenges	[35,36]

***Green synthesis approaches***, aligned with the principles of atom economy aim to maximize the incorporation of all reactant atoms into the final LDH product while minimizing waste and hazardous by-products. These environmentally friendly methods often use mild reaction conditions, renewable or non-toxic precursors, and aqueous solvents to reduce energy consumption and chemical hazards. For LDHs, atom economy is achieved by optimizing stoichiometric ratios during co-precipitation or hydrothermal synthesis to ensure nearly complete metal ion utilization without excess reagents. Additionally, such methods avoid harsh chemicals or organic solvents, enhancing biocompatibility and sustainability which is crucial for biomedical applications. Green synthesis also facilitates scalable, cost-effective production of LDHs with controlled particle size and composition, meeting both environmental and clinical demands [38].

**Post-synthesis methods of LDH nanoplatforms:** Post-synthesis techniques play a crucial role in fine-tuning the properties of LDH nanoplatforms, helping to customize them for particular applications. These methods encompass a range of approaches, but since they primarily focus on modifying or adding functionality to LDHs after their initial creation to achieve specific features, we will focus on discussing just two of these techniques in this section.

***The ion-exchange method*** facilitates the modification of LDHs by replacing interlayer anions with bioactive molecules or drug compounds. This approach involves exposing pre-synthesized LDHs to a mixture comprising the desired anion that interacts with the interlayer space which leads to the exchange of pre-existing anions without disrupting the layered structure [39]. The ion-exchange process enables the incorporation of various curative agents such as anticancer medications, nucleic acids, or imaging agents into LDHs which enhances their biomedical applicability. The efficiency of ion exchange varies on parameters such as ion selectivity, charge density, and interlayer spacing of the LDH material. This method allows the functionalization of LDHs without altering their fundamental structural properties which ensures that their stability and drug-release characteristics remain intact. Compared to other synthesis methods, ion exchange provides a straightforward route for loading bioactive molecules into LDHs to makes it a favorable strategy for drug carriage applications. However, this approach requires careful selection of ion-exchange conditions to prevent unwanted structural changes or aggregation that could affect the function of LDH-based nanoplatforms. It is able to be optimized by carefully controlling pH, temperature, and reaction time. Using mild reaction conditions and gradual ion addition helps maintain the structural integrity of LDH layers. Incorporating stabilizing agents or surfactants can further prevent nanoparticle aggregation. Additionally, performing the exchange in a buffered environment minimizes unexpected pH shifts, preserving the crystallinity and dispersion of the LDH nanoplatforms which is essential for consistent drug loading and release behavior [6]. ***Intercalation*,** similar to ion-exchange but often involves inserting larger molecules or hybrid materials into the LDH interlayer. Intercalation of LDH nanoplatforms is a fascinating process where we insert specific molecules or ions into the spaces between the layers of layered double hydroxides. It all starts with creating the basic LDH structure, often using methods like co-precipitation or hydrothermal treatment. Once we have the clean, pristine LDH, we introduce it to a solution containing the desired molecules, this could be drugs, polymers, or other organic compounds. Under carefully controlled conditions such as pH, temperature, and time, these molecules slip into the interlayer spaces, causing the layers to expand slightly. This expansion allows larger or more functional molecules to be housed neatly within the structure which enhances the material stability, controls how the molecules are released, and enables targeted delivery. The success of intercalation depends on factors like the size and charge of the molecules, as well as how well they fit with the LDH layers. In one study, a tiny fluorescent sensor was developed to detect a key marker (DPA) linked to anthrax bacteria. Mg-Al-LDH was synthesized *via* intercalation technique, which can hold other molecules between its layers. When DPA is present, it triggers the fluorescence from attached ions like terbium (Tb) or europium (Eu), making detection easy and accurate. These sensors can work in liquids, blood serum, or directly on spores, even with the help of a smartphone. This approach shows promise for quick, on-the-spot detection of dangerous pathogens. Overall, intercalation significantly broadens the potential uses of LDHs, especially in drug delivery, catalysis, and sensor technologies, by allowing a wide variety of molecules to be incorporated into their layered structure [40,41].

The choice of synthesis method for LDH-based nanoplatforms significantly influences their biological performance, particularly in terms of cellular uptake, biodegradability, and therapeutic efficacy. Methods like co-precipitation offer high scalability and drug-loading efficiency but often result in broad particle size distributions and lower crystallinity, which can lead to inconsistent cellular uptake and unpredictable biodegradation. In contrast, hydrothermal and sol-gel methods yield highly crystalline and uniform particles enhancing cellular internalization and stability, though their scalability and cost-effectiveness vary. Ion-exchange techniques allow precise incorporation of sensitive bioactives without compromising their activity, yet the multi-step process and moderate yields may hinder reproducibility and large-scale application. Urea hydrolysis, conducted under mild conditions is ideal for heat-sensitive drugs and offers good biocompatibility, though it may result in moderate crystallinity and slower drug release. Microwave-assisted synthesis provides rapid production of crystalline particles with enhanced drug-loading potential and cellular uptake, but its dependence on specialized equipment poses challenges for clinical translation. Overall, each synthesis method imparts distinct physicochemical characteristics to LDH nanoplatforms, which directly imitate their interaction with biological structures and their potential for successful therapeutic application [27,30,33].

### 2.2 Tuning LDH properties and drug loading strategies for cancer therapy

**Fig. 2:**
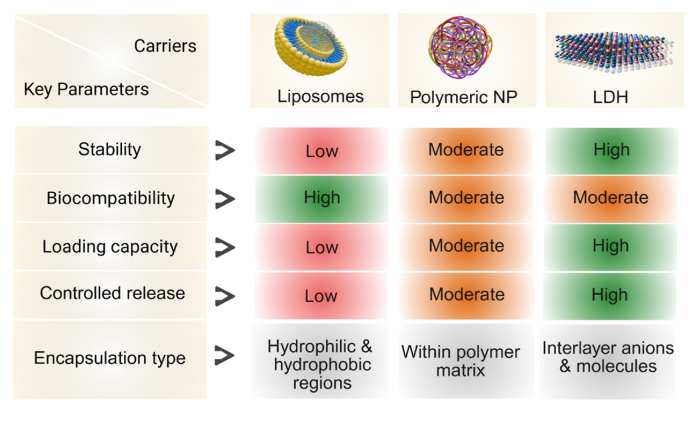
Schematic representation illustrating the superior performance of LDHs compared to other delivery platforms in various biomedical applications. The figure highlights key advantages of LDHs including enhanced drug loading and controlled release capabilities, excellent biocompatibility with minimal cytotoxicity, and superior structural stability under physiological conditions. These advantages contribute to better treatment outcomes, longer circulation times, and fewer side effects and make LDHs a highly promising and versatile option for next-generation drug delivery systems.

The therapeutic performance of LDH-based nanoplatforms in cancer therapy depends on the careful tuning of their physicochemical properties, surface functionalization, and drug loading strategies. LDHs with their intrinsic anion-exchange capacity and two-dimensional lamellar structure provide a robust foundation for accommodating a wide range of therapeutic agents and enabling surface modifications tailored to biomedical applications [4,7]. Adjusting the composition and molar ratios of divalent and trivalent metal cations (e.g., Mg^2+^, Zn^2+^, Ca^2+^, Fe^3+^, Al^3+^) modulates lattice stability, surface charge, and biocompatibility, directly affecting their behavior in the tumor microenvironment (TME) [6,18,42]. For example, Zn^2+^ incorporation can enhance cytocompatibility and introduce intrinsic anticancer activity, while Fe^3+^ containing LDHs impart magnetic properties suitable for imaging-guided and magnetically targeted therapies [43,44] Surface functionalization is critical for improving stability, circulation, and tumor targeting. Hydrophilic polymers such as PEG and biocompatible polysaccharides (e.g., chitosan, hyaluronic acid) enhance colloidal stability, prolong systemic retention, and reduce nonspecific serum interactions [42]. Decorating LDHs with targeting ligands including folic acid, peptides, or monoclonal antibodies, enables receptor-mediated endocytosis and preferential tumor accumulation [45]. Incorporating stimuli-responsive components such as pH-sensitive linkers or redox-cleavable disulfide bonds further refines release profiles, ensuring cargo discharge occurs selectively in acidic or reductive tumor environments [46,47]. Particle size and morphology (typically 50–200 nm) are also crucial, optimizing passive tumor accumulation *via* the enhanced permeability and retention (EPR) effect and influencing cellular uptake and endosomal escape [48]. Drug loading into LDHs can be achieved through multiple approaches depending on the physicochemical nature of the therapeutic cargo. Anionic drugs or prodrugs are commonly intercalated within LDH interlayers *via* ion-exchange or co-precipitation which provides protection and enables slow release, often pH-responsive [32,49]. Neutral or cationic drugs are typically loaded through surface adsorption mediated by non-covalent interactions (e.g., electrostatic forces, hydrogen bonding, van der Waals attractions), while covalent conjugation to functionalized LDH surfaces can improve stability and trigger controlled release in response to intracellular stimuli [50]. These strategies allow precise control over drug stoichiometry, structural stability, and release kinetics with parameters such as layer charge density, interlayer spacing, and particle size distribution playing pivotal roles [2,34]. Table 2 illustrates LDHs alongside other nanocarriers such as liposomes and mesoporous silica nanoparticles with respect to drug-loading efficiency, targeting capabilities, and stages of clinical development. Fig. 2 demonstrates the enhanced effectiveness of LDHs compared to other delivery systems across diverse biomedical applications.

**Table 2:** Comparative analysis of LDHs liposomes, and mesoporous silica nanoparticles (MSNs) in terms of capacity for drug incorporation, targeting efficiency, and clinical development status for cancer therapy applications.

Parameter	LDHs	Liposomes	Mesoporous Silica Nanoparticles (MSNs)	Refs.
Drug-loading capacity	Moderate to High (10–30 wt%) good for ionic drugs and pH-responsive systems	Moderate (5–20 wt%) encapsulates hydrophilic and hydrophobic drugs	High (>30 wt%) large surface area and tunable pore size	[51,52]
Targeting efficiency	Good enhanced by surface modification (e.g., folate, antibody, RGD peptides)	Excellent well-established passive and active targeting	Good easily functionalized with targeting ligands	[53,54]
pH-responsive release	Yes, efficient in acidic tumor microenvironment	Limited typically requires modification for responsiveness	Yes, achieved *via* gatekeepers or cleavable linkers	[49,52]
Biodegradability	Moderate gradual degradation in acidic environments	High Lipid bilayers are enzymatically degraded	Low to moderate non-biodegradable core with potential accumulation	[55,56]
Toxicity concerns	Potential metal ion leaching (e.g., Al^3+^); mitigated by Mg/Ca-based systems	Generally low; FDA-approved for clinical use	Potential long-term toxicity and inflammation due to persistence	[[56], [57], [58]]
Clinical progress	Preclinical stage; limited *in vivo* and safety studies	AdvancedSeveral FDA-approved formulations (e.g., Doxil®, Onivyde®)	Mostly preclinical; a few in early-phase clinical trials	[51,59]
Ease of surface functionalization	Moderate intercalation and surface grafting possible	Highwell-established modification techniques	High amenable to diverse chemical modifications	[[60], [61], [62]]

**Abbreviations**: Arginine-Glycine-Aspartic acid (RGD), Food and Drug Administration (FDA), doxorubicin HCl liposome injection (Doxil®), irinotecan liposome injection (Onivyde®).

Hybrid LDH nanostructures further expand functionality by integrating complementary materials (e.g., gold nanoparticles, mesoporous silica, quantum dots), enabling imaging-guided delivery, photothermal conversion, and synergistic therapies [[63], [64], [65], [66]]. LDH-based nanozymes with tunable redox activity such as PdCu_x_@LDH can generate cascade ROS through oxidase (OXD) and catalase (CAT) activities, disrupting mitochondrial function and enhancing sonodynamic therapy (SDT) efficacy within the complex TME. Optimizing crystal structure, surface activity, and responsiveness to external stimuli maximizes therapeutic outcomes [PdCu_x@LDH]. Finally, modulating crystallinity, layer thickness, and cation composition (e.g., Mg^2+^, Ca^2+^) enhances biodegradability and facilitates renal or hepatic clearance, supporting safer clinical translation [67].

**Fig. 3:**
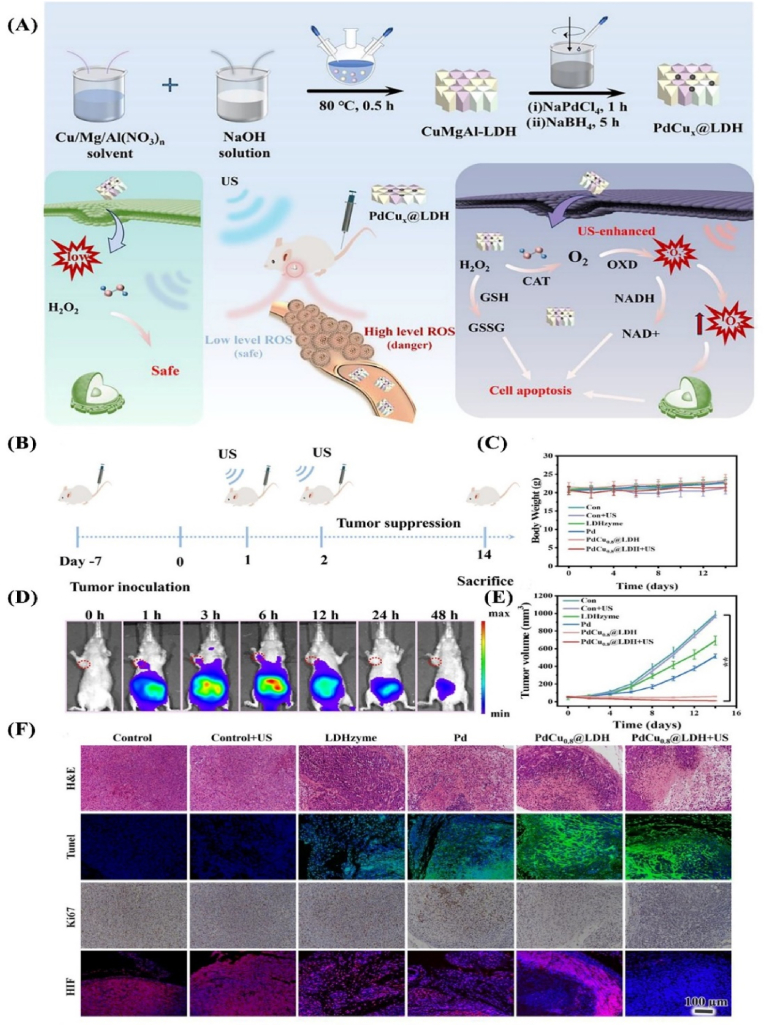
(A) Schematic diagram for the preparation of the PdCux@LDH relieve nanozymes and the proposed antitumor. Mechanism *in vivo* therapeutic performance of PdCux@LDH. (B) Schematic diagram of the timeline of the experiment to investigate the effectiveness of PdCu x@LDH for antitumor therapy. (C) Body weight and (D) *In vivo* fluorescence imaging of the tumor site at several i.v. postinjection time points. (E) tumor volume change curves of HeLa tumor-bearing mice after different therapies (n = 5 per group). (F) H&E, TUNEL, Ki-67, and HIF- 1α antigen staining of tumor tissues taken from the matching mice after 14 days of different therapies. **Abbreviations:** US: ultrasound; Con.: control group. Reprinted with permission from Ref. [67].

Collectively, the rational design of LDHs spanning compositional tuning, targeted surface engineering, and adaptable drug-loading strategies enables precise tumor targeting, controlled release, and integration of multimodal treatments, positioning LDHs as highly versatile nanoplatforms for next-generation cancer therapy [68] Such strategies open new paths for designing nanozymes with precisely controlled activities tailored for targeted and effective cancer treatments (Fig. 3) [67].

## 3 LDHs in therapeutic delivery and multifunctional cancer treatment

**Fig. 4:**
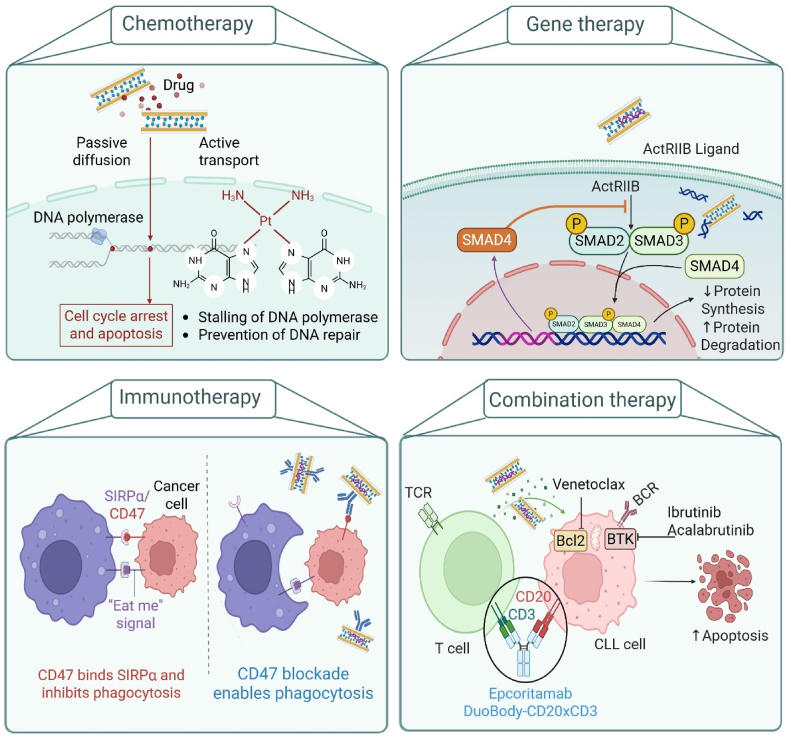
Illustrative summary of LDH nanohybrids and their therapeutic application strategies. Chemotherapy employs cytotoxic drugs; methotrexate (MTX), doxorubicin (DOX), and 5-fluorouracil (5-FU) to kill or inhibit the proliferation of cancer cells. Gene therapy of LDH nanohybrids utilized for diverse cancer therapy strategies involves the delivery of genetic material (DNA or RNA) into target cells to influence gene function and repair genetic defects. Immunotherapy leverages LDH to deliver antigens or immune-stimulating agents like CpG oligodeoxynucleotides to activate and enhance the host immune response against tumors. Combination therapy integrates multiple therapeutic modalities, like co-delivery of anticancer drugs and minor interfering RNA (siRNA), to synergistically target cancer through complementary mechanisms. LDH nanocarriers offer an adaptable platform for improved curative efficacy, localized drug administration, and minimized adverse effects. **Abbreviations:** DNA, Deoxyribonucleic acid; ActRIIB, Activin receptor type IIB; CD47, Cluster of differentiation 47; SIRPα, Signal regulatory protein alpha; TCR, T cell receptor; T cell, T lymphocyte cell; BCR, B cell receptor; BTK, Bruton's tyrosine kinase; Bcl2, B-cell lymphoma 2; CLL Cell, Chronic lymphocytic leukemia cell.

Cancer therapy involves a combination of strategies aimed at eliminating tumor cells while preserving healthy tissues. Despite therapeutic advancements, conventional treatments still face challenges such as drug resistance, systemic toxicity, and insufficient tumor targeting. To address these challenges, advanced nanoplatforms offer new opportunities to enhance therapeutic efficacy. These systems can contribute to cancer treatment by improving drug and gene delivery, enabling light-triggered therapies, and modulating the tumor microenvironment, ultimately leading to tumor suppression and enhanced therapeutic responses [1,2]. Fig. 4 provides a visual summary of the diverse therapeutic strategies enabled by LDH-based nanocarriers, including chemotherapy, gene therapy, immunotherapy, and combination treatments.

### 3.1 Drug delivery and controlled release

LDHs have attracted great attention as nanocarriers for drug delivery and controlled release in cancer therapy due to their unique anion exchange capacity, lamellar structure, pH responsiveness, and biocompatibility. Structurally, LDHs are composed of positively charged metal hydroxide layers, typically consisting of divalent and trivalent metal cations (e.g., Mg^2+^/Al^3+^, Zn^2+^/Fe^3+^), balanced by exchangeable interlayer anions (e.g., CO_3_^2−^, NO_3_^−^, or Cl^−^). This architecture not only facilitates the stable intercalation of a extensive variety of therapeutic agents containing small-molecule drugs, genes, and biomolecules, but also offers a tunable platform for controlled and stimuli-responsive release [1,3,46,69]. The utility of LDHs in drug delivery stems from their ability to encapsulate or intercalate therapeutic agents through physical adsorption, electrostatic interaction, or ion-exchange mechanisms. This allows for a high drug loading capacity and shields sensitive agents from enzymatic degradation or premature release in systemic circulation [13,70]. Additionally, LDHs may also contribute to lysosomal escape through a buffering effect. Their positive surface charge enables favorable interactions with the negatively charged cell membrane. This property promotes efficient cellular internalization, mainly *via* clathrin-mediated endocytosis. After internalization, LDHs accumulate in acidic endosomal compartments. Within these environments, LDHs can partially neutralize protons due to their inherent buffering capacity, promoting osmotic swelling and potential membrane destabilization. This process resembles the proton-sponge effect and may help release the therapeutic cargo into the cytoplasm before lysosomal degradation occurs [[71], [72], [73]].

A key feature of LDH-based drug delivery systems is their pH-sensitive release behavior which holds significant promise for use in treating cancer [18,74]. Acidic conditions are characteristic of tumor microenvironments and intracellular organelles like endosomes and lysosomes (pH ∼4.5–6.5), in contrast to the neutral pH of normal tissues and blood (∼7.4) [62]. Under acidic conditions, the LDH structure undergoes partial dissolution and releases the intercalated or adsorbed drug molecules in a controlled manner [43,75]. This site-specific and pH-triggered drug release minimizes systemic toxicity and enhances local drug concentration at the tumor site, and reduces adverse effects on healthy tissues [69,74]. Zhu et al. [76] investigated the release behavior of 5-aminosalicylate from ZnAl-LDH under simulated intestinal conditions (pH 6.8). Their findings revealed a two-stage release profile: an initial rapid phase due to surface desorption, followed by a slower and sustained release attributed to interlayer diffusion. The cumulative release ranged from 18.8 % to 67.6 % over 12 h, depending on the system composition. Kinetic modeling using the parabolic diffusion equation (R^2^ > 0.97) supports the interpretation that this gradual release reflects not only controlled drug diffusion but also the progressive disassembly of LDH layers over time. These results suggest that drug release kinetics can serve as an indirect indicator of LDH matrix dissolution dynamics. In addition to the influence of pH, the dynamics of interlayer anion exchange also play a critical role in LDH disassembly and drug release behavior. Guo et al. [77] recently clarified through molecular simulations that interlayer anion exchange in LDHs occurs through a two-step process: initially, the structure swells due to water uptake, followed by the gradual replacement of interlayer anions by chloride ions. The efficiency of this exchange is strongly dependent on the strength of interaction between the interlayer anions and the LDH layers; anions with weaker interactions are more easily displaced. In this context, a reduction in the number and stability of hydrogen bonds leads to increased dynamics and greater accessibility for anion exchange. Complementary experimental data from Zhu et al. [76] confirm these findings, showing that the type of interlayer anion significantly influences drug release kinetics. Specifically, LDHs containing carbonate maintain a more stable layered structure and release the drug more slowly under acidic conditions, while nitrate-containing systems exhibit a faster drug release profile.

**Fig. 5:**
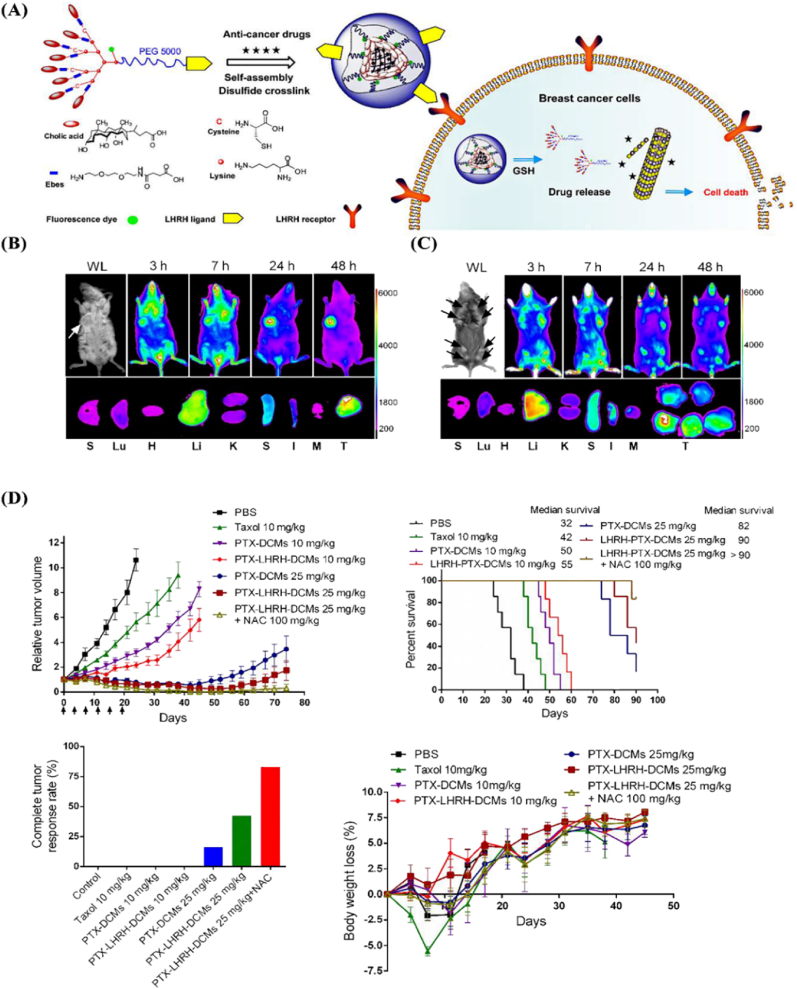
(**A**) A schematic illustrates the breast cancer-targeted delivery of LHRH-DCMs formed *via* self-assembly and oxidation of (D-Lys)-LHRH-conjugated PEG_5_k-Cys_4_-L8-CA8 telodendrimers. These nanoparticles enable precise drug delivery by specifically binding to overexpressed LHRH receptors on breast cancer cells. (**B**) *In vivo* and *ex vivo* near-infrared fluorescence (NIRF) imaging of DiD-labeled LHRH-DCMs showed their distribution in patient-derived xenograft (PDX) tumors and LHRH-DCMs preferentially accumulated in tumors over normal tissues, with confocal imaging confirming deep tumor penetration. (**C**) transgenic (MMTV-PyMT) mouse model of breast cancer revealed sustained accumulation of LHRH-DCMs in spontaneous breast tumors *via* both EPR effect and active targeting, further validated by high tumor uptake 48 h post-injection. (**D**) Anti-tumor efficacy and safety of PTX-LHRH-DCMs were evaluated in an orthotopic breast cancer model. Mice (n = 6–7) received intravenous injections of PBS, Taxol® (10 mg/kg), PTX-DCMs (10 and 25 mg/kg), or PTX-LHRH-DCMs (10 and 25 mg/kg) every four days for six doses. An additional group received 25 mg/kg PTX-LHRH-DCMs with N-acetylcysteine (NAC, 100 mg/kg) 24 h post-injection to trigger drug release at the tumor site. Results included *in vivo* tumor growth inhibition (top-left), survival curves (top-right), complete tumor response rates (down-left), and body weight changes (down-right), with data expressed as mean ± SD (∗*p* < 0.05, one-way ANOVA with Tukey's test). **Abbreviations**: Luteinizing hormone-releasing hormone (LHRH), disulfide crosslinked micelles (DCMs), paclitaxel (PTX), Transgenic murine mammary carcinoma (MMTV-PyMT). Reprinted with permission from Ref. [46].

Furthermore, LDHs can also be engineered for dual or multi-stimuli-responsive release by incorporating external triggers such as redox gradients (e.g., glutathione), temperature, light, or enzymes, further increasing the precision of drug release. For instance, redox-responsive LDHs have been developed using disulfide linkers which are cleaved in the presence of intracellular glutathione, commonly overexpressed in cancer cells. By a research, targeted and stimuli-responsive nanocarriers offer promising strategies for treating triple-negative breast cancer (TNBC). A notable system, luteinizing hormone-releasing hormone (LHRH)-targeted disulfide crosslinked micelles (DCMs) (LHRH-DCMs), employed a micellar carrier composed of poly(ethylene glycol) and dendritic cholic acid that integrated with a redox-sensitive disulfide bond and a (D-Lys)-LHRH peptide for targeted receptor-mediated delivery. These nanoparticles showed high drug loading, glutathione-triggered release, and enhanced uptake by TNBC cells *via* receptor-mediated endocytosis. *In vivo* studies demonstrated superior tumor penetration and therapeutic efficacy of paclitaxel-loaded LHRH-DCMs compared to non-targeted carriers and Taxol® to support their potential for precise TNBC therapy (Fig. 5) [46].

Another key advantage of LDH-based nanocarriers is their capacity for co-delivery of multiple therapeutic agents, enabling synergistic treatment strategies [4,14,78]. LDHs have been effectively applied to co-deliver chemotherapy drugs (e.g., DOX, MTX), gene therapies (e.g., siRNA, plasmid DNA). This co-loading ability provides precise control over the stoichiometry and sequential release of each component, thereby maximizing therapeutic outcomes and potentially overcoming multidrug resistance [14]. The release kinetics and structural stability of LDH nanocarriers can be finely tuned by adjusting formulation parameters. The composition of metal cations, for example, is critical; substituting different divalent and trivalent ions alters interlayer spacing, zeta potential, and dissolution rate of the nanostructure [19,79]. Likewise, the type of interlayer anions (e.g., carbonate, nitrate, chloride) affects intercalation efficiency and governs drug release behavior [17,79]. Particle size and morphology are equally important with nanoscale LDHs exhibiting improved cellular uptake that enhances endocytosis and bioavailability. Surface functionalization strategies including PEGylation, targeting ligands such as folic acid or monoclonal antibodies, and cell-penetrating peptides, significantly enhance LDH biocompatibility, prolong systemic retention, and promote active tumor targeting [64,80]. Surface-engineered LDHs facilitate receptor-mediated endocytosis through ligand receptor interactions, improving selective tumor cell uptake while reducing off-target effects [48,81]. Both *in vitro* and *in vivo* studies confirm that LDH-based drug delivery systems achieve prolonged circulation, high tumor accumulation, and improved therapeutic indices in multiple cancer models [59,82]. Despite these advantages, challenges such as large-scale synthesis, potential long-term toxicity, and immunogenicity remain and require further investigation [18].

**Fig. 6:**
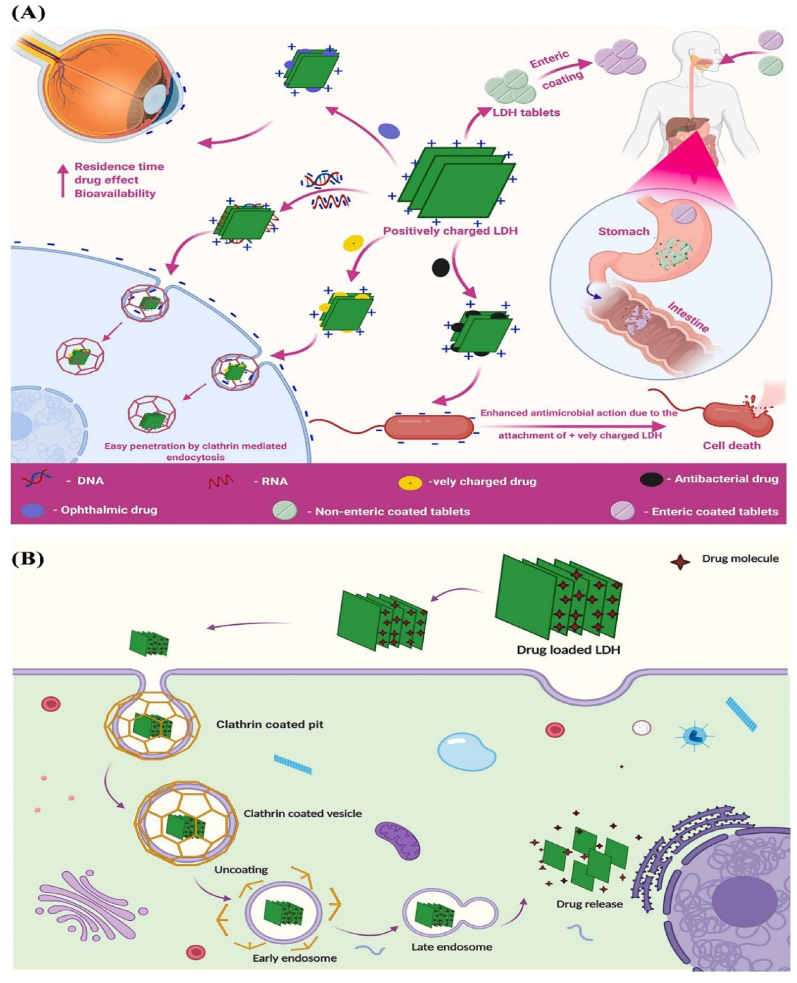
(**A**) The influence of the positive surface charge of layered double hydroxides (LDHs) on their performance in drug and gene delivery systems. The positively charged surface of LDHs facilitates enhanced drug retention by binding to negatively charged ocular tissues, increasing residence time and bioavailability in ocular delivery systems. Additionally, their ability to complex with negatively charged DNA/RNA makes LDHs effective carriers for gene delivery. Due to their alkaline nature and rapid degradation in the stomach, protective enteric coatings are essential for controlling their release and ensuring targeted delivery. (**B**) LDHs enhance drug delivery through their positive surface charge which promotes attachment to negatively charged cellular surfaces and facilitates internalization *via* clathrin-mediated endocytosis. Their high biocompatibility, loading capacity, and stability make LDHs attractive nanocarriers, outperforming other materials such as silica, polymeric nanoparticles, and carbon nanotubes in controlled drug release applications. Reprinted with permission from Ref. [86].

LDH nanoplatforms have emerged as promising non-viral vectors for gene delivery, particularly in the context of RNA interference (RNAi) for cancer therapy. Their unique structure enables the efficient loading and protection of small interfering RNA (siRNA) molecules and facilitate targeted gene silencing within tumor cells [81,83]. The efficacy of LDH nanoparticles in delivering siRNA has been demonstrated in various studies. For example, studies have demonstrated that LDH nanoparticles are capable of strongly associating with siRNA, safeguarding it from degradation, and facilitating its effective delivery to mammalian cells under *in vitro* conditions. The internalization of siRNA-loaded LDH nanoparticles typically occurs through endocytosis, after which the acidic environment within the endosome promotes the dissolution of the nanoparticles and facilitate the release of siRNA into the cytoplasm by aiding endosomal escape. This process results in a significant reduction of target protein expression upon LDH-mediated siRNA transfection [84]. Co-delivery strategies utilizing LDH nanoparticles have also been explored to enhance cancer treatment outcomes. For example, the simultaneous delivery of the anticancer drug 5-fluorouracil (5-FU) and cell death siRNA (CD-siRNA) using LDH nanoparticles has resulted in significantly enhanced cytotoxicity to cancer cell lines compared to single treatments. This synergistic effect is likely due to coordinated mitochondrial damage processes [85]. Optimizing the formulation of LDH-siRNA complexes is crucial for maximizing gene silencing efficiency. Studies have investigated various parameters, including mixing methods, cellular uptake times, and LDH/siRNA mass ratios, to determine optimal conditions for effective gene knockdown. These studies have offered important understanding for developing gene delivery platforms based on LDH materials [81]. Besides, LDH nanoparticles have been integrated into gene-activated scaffolds for tissue regeneration applications, demonstrating their versatility as non-viral vectors. The successful delivery of small nucleic acids, such as siRNA and microRNA mimics, to mesenchymal stromal cells using LDH nanoparticles indicates their potential in regenerative medicine [83]. Fig. 6 presents the positive surface charge of LDHs significantly enhances their effectiveness in drug and gene delivery. In ocular systems, this charge promotes strong binding to negatively charged tissues, increasing drug retention, residence time, and bioavailability. For gene delivery, LDHs form stable complexes with negatively charged DNA/RNA, facilitating protection and cellular uptake *through* clathrin-mediated endocytosis. Their instability in acidic environments necessitates enteric coatings for targeted oral delivery. Compared to other carriers like silica or polymeric nanoparticles, LDHs offer superior biocompatibility, loading capacity, and controlled release, that make them highly effective therapeutic nanoplatforms [86].

### 3.2 Photothermal and photodynamic therapies

**Fig. 7:**
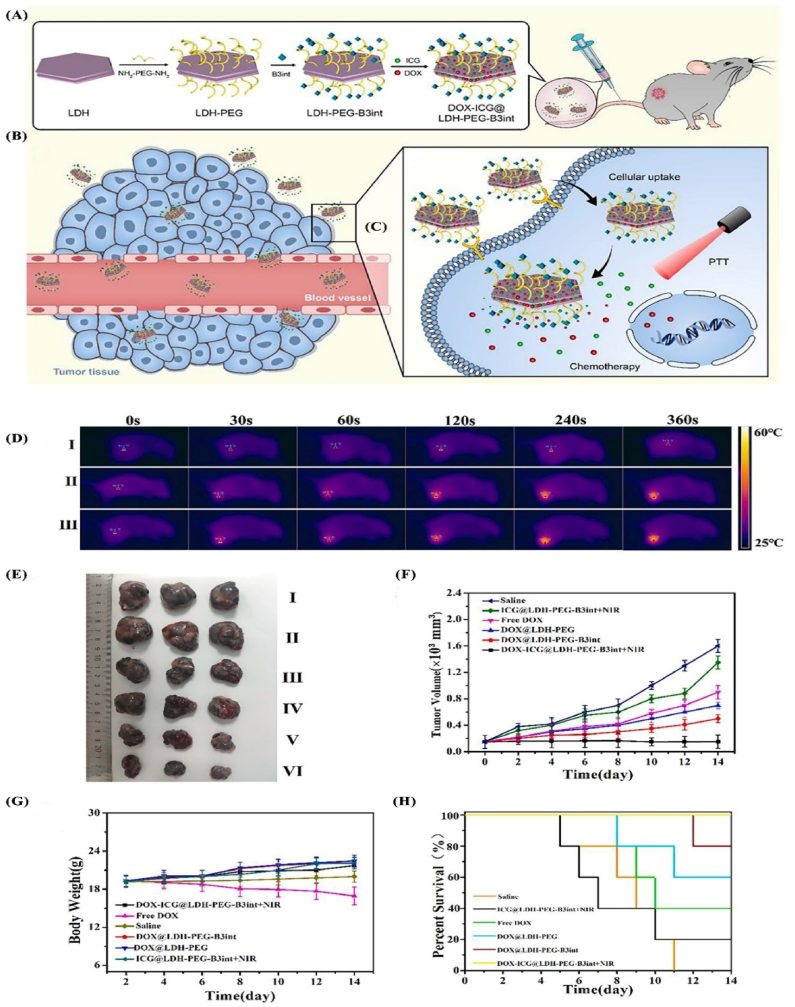
(**A**) Synthesis process of DOX-ICG@LDH-PEG-B3int NPs. (**B**) Targeted accumulation of NPs at the tumor site. (**C**) DOX and ICG release in the tumor environment. (**D**) Thermal profiles of mice with B16 tumors recorded subsequent to diverse treatment approaches. (I: Saline; II: Free ICG; III: DOX-ICG@LDH-PEG-B3int NPs). (**E**) After 14 days of therapeutic intervention, tumors were isolated and subsequently imaged. (**F**) B16 tumor growth curves following IV administration of different formulations. (**G**) Variations in body weight observed throughout the 14-day experimental period. (**H**) Kaplan–Meier survival curves (n = 8). All evidence confirmed the high safety and biocompatibility of DOX-ICG@LDH-PEG-B3int as a chemo-photothermal nanodrug. **Abbreviations:** Indocyanine green (ICG), doxorubicin (DOX), Polyethylene glycol (PEG), Indocyanine green (ICG), near-infrared (NIR). Reprinted with permission from Ref. [7].

LDH nanoplatforms have attracted significant interest as light-responsive agents in cancer therapy, particularly in photothermal therapy (PTT) and photodynamic therapy (PDT), where targeted irradiation is used to induce cancer cell destruction [6,7,[87], [88], [89], [90], [91], [92]]. In PTT, LDH nanoparticles are designed to absorb near-infrared (NIR) light and convert it into localized heat, producing hyperthermia that triggers apoptosis. For instance, ferrous ion-doped LDH nanoparticles (Fe-LDH/DOX) achieved a high photothermal conversion efficiency of 45.67 %, and their combination of PTT and chemotherapy led to effective tumor suppression in 4T1-bearing mice [6]. Similarly, LDH nanoplatforms co-loaded with doxorubicin (DOX) demonstrated pH-responsive and NIR-triggered drug release, significantly enhancing antitumor efficacy *in vitro* and *in vivo* (Fig. 7) [7].

In PDT, LDH nanohybrids improve the solubility and photostability of photosensitizers, addressing common limitations in their clinical application. Curcumin, a natural compound with potent anticancer activity but poor water solubility was successfully intercalated into LDH layers *via* co-precipitation, yielding a 50 % loading capacity and superior photodynamic activity against MDA-MB-231 human breast cancer cells compared with free curcumin [93]. Combined PTT and PDT strategies have also been developed to maximize therapeutic performance. Dihydroartemisinin (DHA)-loaded MnMgFe-LDH nanocomposites exhibited a photothermal conversion efficiency of 10.7 % and produced reactive oxygen species (ROS) through Fenton-like reactions, achieving notable tumor inhibition [11]. Furthermore, LDH nanoparticles coated with polydopamine (PDA) were engineered for synergistic chemodynamic and photothermal therapy, where acid-triggered DOX release and elevated ROS production produced marked anticancer effects [5].

Structural and functional characterization of these LDH-based systems commonly involves X-ray diffraction (XRD) to verify crystallinity, transmission electron microscopy (TEM) to analyze morphology, Fourier-transform infrared spectroscopy (FTIR) to confirm surface modification, and Brunauer–Emmett–Teller (BET) analysis to determine surface area and porosity, all of which are essential for optimizing their light-activated therapeutic performance [87].

### 3.3 Theranostic and imaging-guided applications

Beyond light-based therapies, LDH nanoplatforms have gained prominence in cancer theranostics, where therapeutic and diagnostic capabilities are seamlessly integrated into a single system [44,94]. Their positively charged lamellar structure enables the intercalation of diverse anionic drugs, nucleic acids, and imaging agents, supporting multifunctional and precisely targeted cancer management. For instance, Gd^3+^ doped LDH nanosheets co-loaded with DOX and ICG have demonstrated dual-modal imaging functions, combining near-infrared fluorescence (NIRF) and T_1_-weighted MRI, enabling accurate tumor localization and real-time tracking of drug distribution for improved therapeutic outcomes [95]. Similarly, manganese-doped LDH nanoparticles (Mn-LDH NPs) offer significant T_1_-weighted MRI contrast enhancement and effective photothermal therapy (PTT) under NIR laser irradiation with excellent biodegradability and biosafety for imaging-guided treatment [55]. Incorporation of ultrasmall iron oxide nanoparticles stabilized by LDH and functionalized with hyaluronic acid (HA) further enables targeted T_1_-weighted MRI and pH-responsive drug release, achieving enhanced tumor penetration and selectivity in CD44-overexpressing cancers [44].

LDH nanohybrids also hold promise in gene therapy, where intercalation of plasmid DNA or small interfering RNA (siRNA) protects genetic cargo from enzymatic degradation and enhances cellular uptake, resulting in effective gene silencing and therapeutic benefits in cancer models. Multifunctional LDH-based systems have been engineered to co-deliver chemotherapeutic agents and immune checkpoint inhibitors, potentiating antitumor immune responses and improving treatment efficacy [96]. These combined therapeutic and diagnostic functionalities, reinforced by structural tunability and high biocompatibility, position LDH-based nanoplatforms as next-generation theranostic agents with significant potential to advance precision oncology and personalized cancer therapy [40,55,94]. Table 3 summarizes LDH-based nanoplatforms developed for cancer therapy with highlighting their use in delivering various therapeutic agents, surface modifications, targeting strategies, and observed treatment outcomes. This overview emphasizes the potential of LDHs in enhancing cancer treatment effectiveness.

**Table 3:** Overview of LDH-based nanoplatforms for cancer therapeutic applications.

LDH Composition	Therapeutic Agent	Synthesis/Functionalization Method	Therapy Type	Key Insights	*In Vivo* Efficacy	Toxicity Data (IC_50_ for Normal Cells)	Ref.
MgAl	siRNA	Co-precipitation	Gene therapy	Effective gene silencing *via* LDH-siRNA carriers	Gene knockdown efficiency confirmed *in vivo*	Minimal cytotoxicity to normal cells	[96,97]
MgAl	DNA, FITC, ATP	Ion-exchange	Gene delivery	High transfection efficiency	High gene expression *in vivo*	–	[98,99]
MgAl	siRNA, FITC	Co-precipitation, silane coupling	Gene therapy & Imaging	FA-conjugated targeted delivery; *in vivo* siRNA therapy	Tumor growth inhibition observed in mice	IC_50_ > 100 μg/mL for normal cells	[45,100]
MnAl	siRNA, Mn^2+^	Co-precipitation, self-assembly	Gene therapy, MRI	T_1_-weighted MRI signal enhancement	Effective imaging & therapeutic silencing *in vivo*	Low toxicity; IC_50_ > 75 μg/mL	[82,101]
MgAl	MTX	Co-precipitation	Chemotherapy	Demonstrated efficacy in tumor inhibition	Tumor volume reduced by ∼60 % in mice	IC_50_ ∼10 μM for normal fibroblasts	[102,103]
LiAl	Dox	Co-precipitation	Chemotherapy	DFT-based interaction with DOX; sustained release; intracellular trafficking *via* lysosomes	Significant tumor inhibition in melanoma-bearing mice; good *in vivo* compatibility	Negligible systemic toxicity observed *in vivo*	[104]
MnFe	MTX, Mn^2+^	Co-precipitation	Chemotherapy, MRI	First application of MnFe-LDH system	Tumor regression in xenograft models	Slightly increased toxicity vs. free MTX	[94,105]
MgAl	DOX, PAA	Co-precipitation, ion-exchange	Chemotherapy	DOX activity enhanced with polymer coating	70 % tumor growth inhibition in mice	IC_50_ > 50 μg/mL in normal liver cells	[58,102,106]
NaCa	DAC	Urea hydrolysis	Chemotherapy	NaCa-LDH improved DAC utilization	Prolonged survival in murine melanoma model	Moderate toxicity; IC_50_ ∼12 μM	[39,49]
MgAl	CpG, OVA	Co-precipitation	Immunotherapy	Route-specific delivery improved immune activation	2–3 × increase in IFN-γ secretion *in vivo*	Negligible systemic toxicity	[107]
MgAl	microRNA-155	Co-precipitation	Immunotherapy	Combinational approach boosted immune responses	Enhanced cytokine expression, tumor rejection	Low systemic toxicity	[108]
MgAl	5-FU	Co-precipitation, ion-exchange	Combination (Gene + Chemo)	Synergistic therapeutic effect *via* dual delivery	Tumor volume reduced >65 % *in vivo*	IC_50_ ∼15 μM for normal epithelial cells	[68,109]
MgCuAl	DC, LOX	Co-precipitation	Chemodynamic + Immunotherapy (Combination)	Dual regulation of lactate and redox metabolism; synergy between CDT and immunotherapy; cascade effect	Tumor inhibition ∼79.3 %; increased CD4^+^/CD8^+^ T cell infiltration; cytokine (IFN-γ) upregulation	Minimal toxicity *in vivo*; no major organ damage	[110]

**Abbreviasions**: small interfering RNA (siRNA), fluorescein isothiocyanate (FITC), adenosine triphosphate (ATP), methotrexate (MTX), doxorubicin (DOX), poly(acrylic acid) (PAA), dacarbazine (DAC), CpG oligodeoxynucleotides (CpG), ovalbumin (OVA), 5-fluorouracil (5-FU), chemodynamic therapy (CDT), lactate oxidase (LOX), diclofenac (DC), half maximal inhibitory concentration (IC_50_), density functional theory (DFT), interferon-gamma (IFN-γ).

### 3.4 Synergistic and combination treatment strategies

**Fig. 8:**
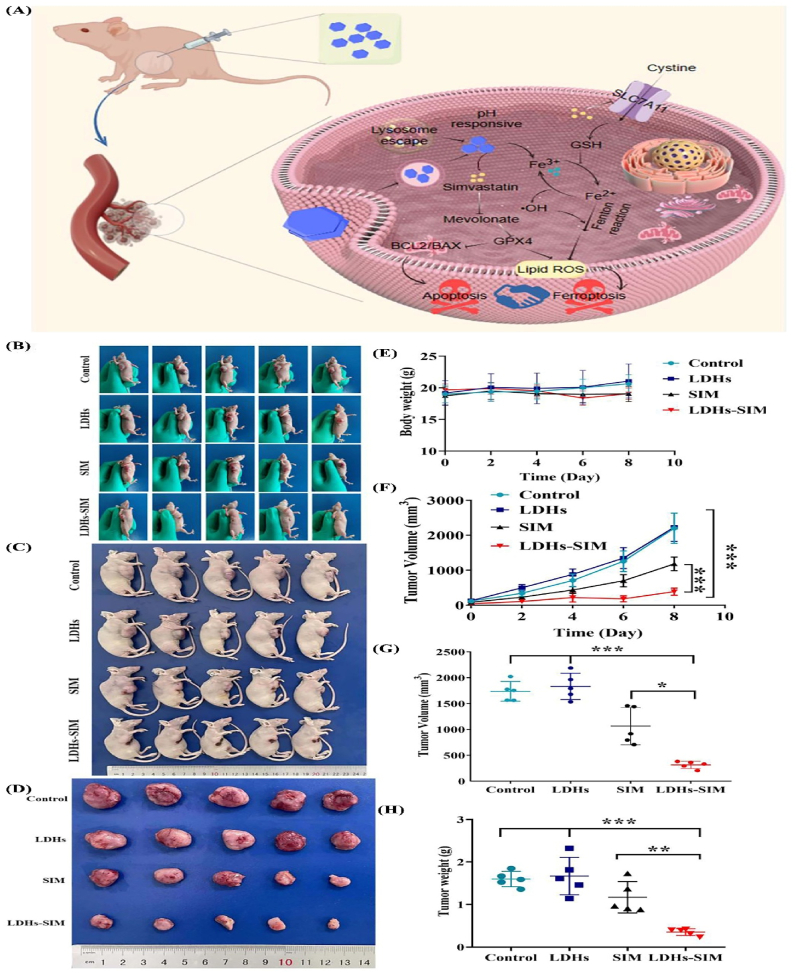
(**A**) schematic of the Mg-Fe-LDHs-SIM nanodrug system for breast cancer treatment shows that once taken up by cancer cells, Fe^3+^ ions are released to generate hydroxyl radicals, increasing ROS levels. Simvastatin (SIM) released from the nanocarrier inhibits GPX4 *via* the mevalonate pathway and downregulates SLC7A11 and reduced GSH levels and triggering ferroptosis. Representative images and analysis of subcutaneous tumor development in nude mice across different treatment groups. (**B**) Photograph of tumors in each group on day 8 post-implantation. (**C**) Tumor growth progression following treatments. (**D**) Photographs of excised tumors illustrating their anatomical features. (E) Body weight changes over time in the treated mice. (**F**) Tumor volume growth curves during the treatment period. (**G**) Tumor volumes measured after dissection. (**H**) Tumor weights estimated post-mortem. Data are shown as mean ± SD for five mice per group. Statistical significance is indicated as follows: ∗*p* < 0.05, ∗∗*p* < 0.01, ∗∗∗*p* < 0.001.Reprinted with permission from Ref. [111].

Combination therapies leveraging multifunctional LDH-based nanoplatforms have emerged as a prospective intervention to increase the therapeutic impact of cancer therapies by integrating multiple therapeutic modalities into a single system. These nanoplatforms are designed to exploit the synergistic effects of combined therapies such as chemotherapy, PTT, PDT, immunotherapy, and ferroptosis induction, thereby overcoming the limitations associated with monotherapies [7,46,111]. One notable example involves the development of functionalized LDH-based nanostructures with the B3int peptide which targets integrin *αvβ*3 overexpressed in certain cancer cells. These nanoparticles co-deliver DOX and ICG facilitated a synergistic PTT/chemotherapy approach. The system exhibited high drug loading capacity and significant photothermal conversion efficiency with pH-responsive and NIR-triggered drug release mechanisms. *In vitro* and *in vivo* studies have demonstrated that this co-delivery system significantly enhances antitumor activity compared to single-agent therapies [7]. Another innovative approach utilizes LDH nanoparticles to co-deliver methotrexate (MTX) and 5-fluorouracil (5-FU), two chemotherapeutic agents commonly used in combination therapy. The resulting nanohybrids exhibit a house-of-cards-like structure that accommodates both drugs and enhanced therapeutic efficacy in HeLa cells compared to individual drug treatments or their mixtures. This suggests that multidrug-incorporated LDH nanohybrids could serve as effective carriers for combination chemotherapy [13]. LDH-based nanoplatforms have also been explored for initiating ferroptosis, a type of programmed cell death that involves lipid peroxidation dependent on iron accumulation. For instance, in Fig. 8 simvastatin-loaded LDH nanoparticles have been shown to induce both apoptosis and ferroptosis in breast cancer cells. The system promotes the accumulation of Fe^2+^ ions and ROS within cells while inhibiting glutathione peroxidase 4 (GPX4) and SLC7A11 expression and enhanced anticancer effects with minimal systemic toxicity [111].

In immunotherapy, in conjunction with PD-L1 blockade and PTT, ultra-thin LDH nanosheets have been engineered to deliver siRNA targeting the intracellular immune checkpoint NR2F6. This multifunctional nanoplatform effectively modulated the tumor microenvironment and enhanced T-cell infiltration and antitumor immunity in hepatocellular carcinoma models [112]. Also, LDH-based nanocomposites have been designed for combined PDT and chemotherapy. For example, zinc phthalocyanines (ZnPc) intercalated into LDH galleries, followed by DOX loading on the surface and created a system that enhances PDT efficiency while providing chemotherapeutic effects. Studies conducted *in vitro* using KB cells revealed that the combined treatment exhibited enhanced anticancer efficacy and superior biocompatibility compared to the use of either ZnPc or DOX individually [8,113].

## 4 Preclinical and clinical evaluations

### 4.1 *In vitro* studies: efficacy and cytotoxicity

*In vitro* studies have demonstrated the efficacy and cytotoxicity profiles of LDH-based nanoplatforms in cancer therapy. For instance, LDH nanoparticles loaded with disuccinatocisplatin (DSCP) exhibited significantly enhanced cytotoxicity against A2780 ovarian cancer cells compared to free DSCP with an IC_50_ value of 0.34 μM versus 1.8 μM, respectively. Notably, these nanoparticles showed reduced toxicity toward normal cells, that indicated a favorable therapeutic index [2]. Similarly, MTX and 5-FU were co-incorporated into LDH nanoparticles that resulted in a nanohybrid (MFL) that demonstrated superior anticancer efficacy in HeLa cells in contrast to either separate drug-loaded LDHs or unencapsulated drugs. This suggested a synergistic effect facilitated by the LDH carrier [114]. Additionally, LDH nanoparticles have been utilized to deliver microRNA-30a (miR-30a) to breast cancer cells and leaded to suppressed cell proliferation with accumulation of cells in the G0/G1 phase. This indicated the potential of LDH-based systems in gene therapy applications [82]. Moreover, LDH nanoparticles loaded with simvastatin (LDHs-SIM) have been shown to induce both apoptosis and ferroptosis in breast cancer cells led to improved therapeutic outcomes with reduced systemic side effects [111].

### 4.2 *In vivo* studies: pharmacokinetics and tumor targeting

The *in vivo* evaluation of LDH-based nanoplatforms has demonstrated their ability to improve pharmacokinetic profiles and enhance tumor-targeting capabilities. These nanocarriers exhibit favorable biodistribution, prolonged circulation times, and increased tumor accumulation which are critical for effective cancer therapy [7]. In a study involving MTX-loaded LDH nanoparticles, the hybrid system displayed a biexponential decline in plasma MTX levels similar to free MTX. However, the MTX–LDH nanohybrids achieved significantly higher tumor suppression in human osteosarcoma-bearing mice, attributed to improved MTX delivery to tumor tissues and reduced off-target distribution, resulting in enhanced efficacy and decreased systemic toxicity [59].

Surface modifications responsive to the tumor microenvironment have been explored to further increase tumor accumulation. LDH nanoparticles coated with pH-sensitive, charge-convertible polymers showed reduced uptake by normal cells at physiological pH but increased uptake by tumor cells under acidic conditions. *In vivo* MRI confirmed effective tumor accumulation of these nanohybrids, demonstrating their potential as versatile delivery systems for enhanced antitumor treatment [74]. Biomimetic strategies such as cloaking LDH nanoparticles with cancer cell membranes have been employed to facilitate homologous targeting and avoid immune surveillance. In colorectal carcinoma models, membrane-coated LDH nanocomposites loaded with ICG and paclitaxel exhibited enhanced tumor accumulation and significant tumor growth inhibition under laser irradiation with no detectable adverse effects in other organs [8].

LDH systems have also been applied for targeted gene delivery. For instance, LDH was used to deliver the tumor-suppressor miRNA, miR-30a. The resulting LDH@miR-30a complex achieved efficient miRNA loading, lysosomal escape, and intracellular retention in SKBR3 breast cancer cells for over 24 h. *In vitro*, LDH@miR-30a inhibited proliferation, induced G0/G1 arrest, and suppressed migration and invasion by targeting SNAI1 and modulating EMT. *In vivo*, LDH@miR-30a significantly reduced tumor growth in nude mice without affecting major organs, suggesting its potential as a safe and effective therapy for breast cancer [82].

**Fig. 9:**
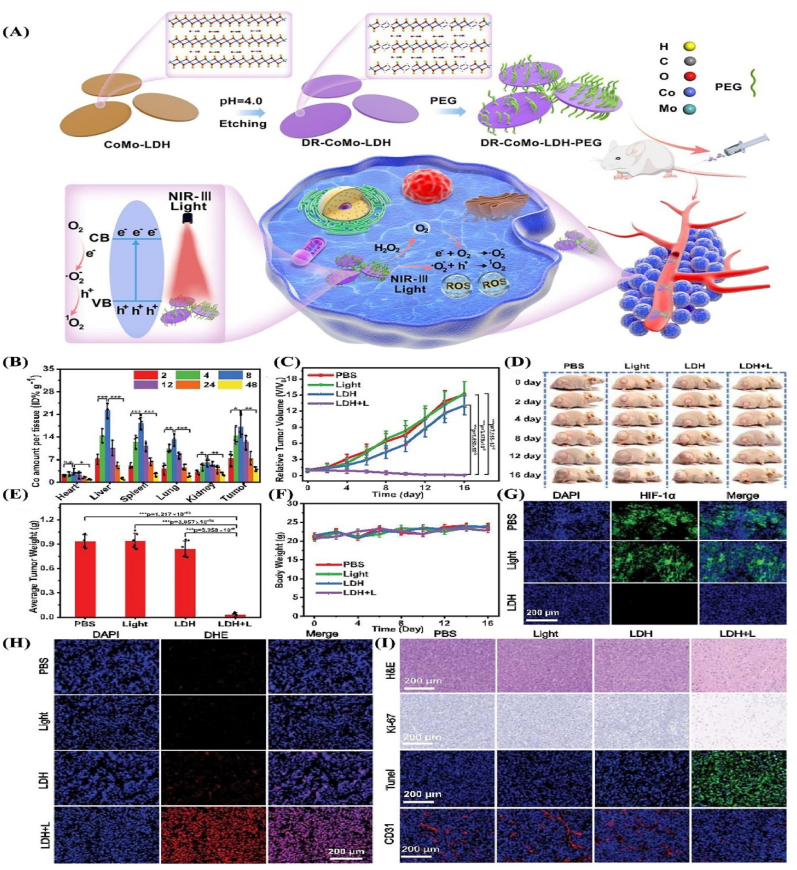
(**A**) Schematic representation of the defect engineering process for CoMo-LDH nanosheets, PEG surface modification, and their application in NIR-III photodynamic therapy (PDT). Initially, CoMo-LDH nanosheets are impressed at pH 4.0 for 2 h to introduce structural defects, followed by PEGylation to produce defect-rich DR-CoMo-LDH-PEG nanosheets. Upon exposure to a 1567 nm laser, these nanosheets generate substantial reactive oxygen species (ROS), enabling effective tumor ablation. (**B**) Tracking the biodistribution of DR-CoMo-LDH-PEG in mice through measurement of cobalt (Co) levels at different time intervals after injection. Data are shown as mean values ± SD (n = 3). Statistical analysis was achieved *via* one-way ANOVA. ∗*p* < 0.05, ∗∗*p* < 0.01, ∗∗∗*p* < 0.001. (**C**) Progression of tumor size in mice following different treatment regimens. Data are shown as mean values ± SD (n = 6). Statistical analysis was completed *via* one-way ANOVA. ∗∗∗*p* < 0.001. (**D**) Representative images of treated mice at different time points and (**E**) the corresponding average tumor weights measured on Day 16. Data are presented as mean values ± SD (n = 6). Statistical analysis was presented *via* oneway ANOVA. ∗∗∗*p* < 0.001. (**F**) Variation in body weight of 4T1 tumor-bearing mice following various treatment regimes. Data are presented as mean values ± SD (n = 6). (**G**) HIF-1α, (**H**) DHE, and (**I**) H&E, Ki-67, TUNEL and CD31 histological staining analysis of tumor sections from different groups of mice following 16 days of treatment; 1) PBS, 2) 1567 nm laser (0.5 W cm-2 for 6 min), 3) DR-CoMo-LDH-PEG, 4) DR-CoMo-LDH-PEG + 1567 nm laser. Each test was conducted in triplicate. Reprinted with permission from Ref. [115].

**In** Fig. 9, defect-rich CoMo-LDH and NiMo-LDH nanosheets produced *via* acid etching of hydrothermally synthesized 2D structures, exhibited favorable pharmacokinetics with prolonged blood circulation and enhanced tumor accumulation after PEG modification. The engineered surface defects markedly improved intratumoral retention, resulting in up to a ∼97-fold increase in ROS generation within tumor sites compared to pristine counterparts under NIR-III (1567 nm) laser irradiation. This enhanced tumor-specific activation underscores the potential of defect-engineered LDHs as highly effective inorganic PSs for targeted NIR-III–mediated therapy [115].

LDHs undergo gradual biodegradation in acidic intracellular compartments (e.g., endosomes and lysosomes), releasing constituent metal cations and intercalated agents [55,116]. The extent of this process depends on particle size, composition (e.g., MgAl *vs*. ZnFe LDHs), and surface modifications. A concern for long-term use is metal ion leaching, especially in Al-containing LDHs (e.g., MgAl-LDHs), where Al^3+^ release under acidic or oxidative conditions may pose neurotoxicity and systemic toxicity risks. Other metal-based LDHs (Zn^2+^, Fe^3+^, Co^2+^) may also exert cytotoxic effects at high concentrations or with prolonged exposure [44,69,75]. Strategies to mitigate these risks include substituting trivalent cations with biocompatible divalent ions (e.g., Mg^2+^, Ca^2+^) [31,32,116] and applying surface functionalizations such as PEGylation, phospholipid coating, or silica encapsulation which minimize ion exposure and enhance systemic stability [64,80]. Controlled-release formulations that exploit tumor-specific stimuli (e.g., pH, redox gradients) can further localize drug release and reduce off-target effects [46]. Incorporating LDHs with biopolymers (e.g., chitosan, alginate) or carbon-based materials has also improved nanoparticle stability and reduced metal ion exposure, making these hybrid systems promising candidates for safe and effective cancer therapy [117]. Table 4 shows *in vivo* toxicity in terms of various common parameters.

**Table 4:** Summary of *in vivo* toxicity, clearance mechanisms, and biodegradation risks of LDH nanomaterials.

Parameter	Findings	Notes/Examples	Ref.
Organ-specific accumulation	Liver, spleen, and lungs show highest accumulation due to RES uptake	Similar to other nanoparticles; hepatic accumulation most common	[118]
Immune response	Minimal acute inflammation; cytokine levels (IL-6, TNF-α) slightly elevated at high doses	Surface modification (e.g., PEGylation) reduces immune recognition	[118]
Clearance mechanism	Both renal and hepatic clearance observed depending on size and surface charge	<10 nm: renal excretion dominant; >50 nm: hepatobiliary route	[119]
Biodegradation	Gradual disintegration in acidic tumor microenvironment or lysosomes	Useful for drug release; complete degradation varies (days to weeks)	[120]
Metal ion leaching	Aluminum (Al^3+^) and other trivalent cations may leach under acidic or oxidative conditions	Risk of neurotoxicity with Al^3+^ if not properly formulated	[121]
Mitigation strategies	Use of Mg^2+^/Ca^2+^-based LDHs; surface coatings (e.g., silica, PEG); hybridization with biopolymers	Reduces systemic toxicity and improves biodegradability	[120]

**Abbrevisions:** eticuloendothelial system (RES), Polyethylene glycol (PEG), Interleukin-6 (IL-6), Tumor necrosis factor-alpha (TNF-α).

## 5 Advantages, challenges and limitation of LDH nanoplatforms

LDH nanoplatforms have gained significant attention in cancer therapy due to their inherent biocompatibility and stability, stemming from their composition of naturally occurring metal ions and hydroxide layers that are generally well-tolerated by biological systems [39]. Their structural versatility permits incorporation of numerous therapeutic agents while maintaining compatibility with human tissues, with their layered structure providing a protective environment that prevents premature breakdown and ensures controlled release of encapsulated drugs. Recent studies have demonstrated enhanced therapeutic efficacy, such as doxorubicin-loaded MgAl-LDHs showing improved tumor suppression in H22 tumor-bearing mice compared to free doxorubicin, attributed to sustained release and targeted delivery facilitated by the LDH matrix. Surface modification strategies using biocompatible polymers like PEG or natural biomolecules such as bovine serum albumin have further enhanced colloidal stability, prolonged circulation time, and reduced immunogenicity while enabling targeted delivery through attachment of cancer cell-specific ligands. Beyond drug delivery, LDHs have shown promise in diagnostic applications by stabilizing imaging agents like ultrasmall iron oxide nanoparticles for enhanced MRI contrast capabilities. Despite these advantages, LDH nanoplatforms face significant challenges in overcoming biological barriers, though they possess unique properties to address limitations such as immune clearance, poor tumor targeting, and inefficient intracellular delivery [40,122]. Biomimetic strategies, including coating LDH nanoparticles with cancer cell membranes, have demonstrated reduced immune recognition and improved targeting, as shown by cancer cell membrane-coated LDH nanosheets loaded with indocyanine green and paclitaxel-albumin complexes that achieved efficient tumor accumulation and growth inhibition upon near-infrared irradiation [8]. Surface functionalization with targeting ligands like folic acid enhances cancer cell specificity, while pH-responsive designs enable preferential drug release in acidic tumor microenvironments, with doxorubicin and indocyanine green-loaded LDH nanoparticles demonstrating enhanced anti-tumor efficacy through pH-responsive and near-infrared-triggered drug delivery [7]. Co-delivery systems incorporating multiple therapeutic agents, such as methotrexate and 5-fluorouracil encapsulated in LDH nanohybrids, have shown enhanced anticancer activity compared to monotherapy treatments by overcoming multidrug resistance [13].

The scalability of production presents critical challenges for transitioning LDH nanoplatforms from laboratory research to clinical applications, particularly in maintaining consistent particle size and morphology control during large-scale synthesis [39]. Precise control of synthesis conditions including temperature, pH, and reactant concentrations is essential, as slight variations can lead to profound differences in end product characteristics, exemplified by Cu–Al LDH nanoparticle synthesis for doxorubicin delivery requiring specific conditions for enhanced safety profiles and biocompatibility [14]. Reproducibility of functionalization processes, such as conjugation with targeting ligands like folic acid or hyaluronic acid, must be maintained during scale-up to ensure consistent therapeutic efficacy and biodistribution. Complex co-delivery systems, like 5-fluorouracil and siRNA using MgAl-LDH nanoparticles, require meticulous optimization to maintain desired drug loading ratios and release profiles during scaling, while integration of imaging probes for theranostics adds additional complexity requiring uniform distribution and stability within the LDH matrix [44,123].

The transformation from laboratory to industrial-scale production faces several critical limitations, including high costs associated with expensive reagents and energy-intensive procedures such as hydrothermal treatment and controlled co-precipitation [63]. Maintaining batch-to-batch consistency remains challenging, as small changes in synthesis parameters can result in inconsistencies in particle size, surface charge, and drug loading, directly affecting performance and safety profiles [18,63,124]. Long-term storage and stability issues arise from LDH nanoparticles' susceptibility to aggregation, structural degradation, or phase transformation over time, potentially affecting bioactivity and safety. Key safety concerns include uncontrolled ion leaching that risks off-target toxicity and disruption of systemic metal ion balance, with common MgAl-LDHs potentially releasing neurotoxic Al^3+^ under acidic or oxidative conditions. Tumor microenvironment heterogeneity limits predictable ROS generation, while structural instability in physiological fluids and cumulative metal ion deposition pose chronic toxicity risks, compounded by incomplete pharmacokinetic and biodegradation data that hinder regulatory approval [31].

To address these limitations, microfluidic synthesis has emerged as a promising solution, offering precise control over reaction parameters and enabling continuous, reproducible, and scalable production of LDH nanoparticles [[125], [126], [127]]. These platforms facilitate fine-tuning of critical characteristics such as size, shape, and drug loading while minimizing reagent use and energy consumption, thereby reducing costs and improving environmental sustainability. Combined with surface engineering and stimuli-responsive designs, microfluidic approaches can precisely control composition, stabilize structures, and enable targeted, predictable metal ion release for safer and more effective therapies. The development of standardized protocols integrated with real-time monitoring systems and quality control procedures will be essential for maintaining nanoparticle property homogeneity during mass production, while environmentally friendly synthesis methods may provide more sustainable and scalable approaches for bringing LDH-based cancer nanomedicines closer to clinical and commercial viability.

## 6 Future perspectives in LDH-based cancer therapy

The future of LDH-based cancer therapy is characterized by remarkable advances in multifunctional nanoplatforms that integrate therapeutic and diagnostic capabilities to overcome monotherapy limitations [13,128]. Recent developments have demonstrated the potential of LDH nanocarriers for synergistic photothermal therapy (PTT) and chemotherapy, exemplified by DOX and ICG co-loaded nanoparticles with targeting peptides that exhibit high drug-loading capacity and significant photothermal conversion efficiency [7], while DOX-loaded Fe-doped LDH nanoparticles engineered for MRI-guided photochemotherapy show pH-responsive drug release and effective tumor suppression [88]. The integration of PTT with chemodynamic therapy through platforms like DOX-loaded Cu–Al LDH nanoparticles with polydopamine coating has achieved Fenton-like catalytic activity for ROS production and synergistic CDT–PTT effects [5], with long-term implications including improved bioavailability and sustained antitumor activity, as demonstrated by PPT–LDH hybrids achieving 46.4 % tumor suppression rates [3].

These systems enable combination therapies that reduce drug dosages and minimize side effects, such as MTX and 5-FU co-delivery within LDH matrices [13], while natural bioactive compounds like curcumin and gallic acid incorporated into LDH frameworks offer sustained cancer treatment by inhibiting cell migration and demonstrating significant cytotoxicity [70]. The evolution toward theranostics through incorporation of MRI and fluorescence imaging agents enables simultaneous therapy and real-time treatment monitoring [16], with immunotherapy applications showing promise through platforms like FeOOH/Cu-LDH loaded with ganetespib that induce immunogenic cell death and complete tumor regression [129]. Hybridization with other nanomaterials maximizes therapeutic effectiveness through synergistic combinations, including Fe_3_O_4_-LDH nanocomposites for magnetically guided drug delivery with pH-responsive release [78], CuS nanodots grown on LDH nanoplates for dual-mode photothermal and photodynamic therapy [4,88], and NiFe-LDH/Ti_3_C_2_ MXene nanocomposites that demonstrate synergistic chemotherapy, PTT, and CDT effects [9]. The integration with wearable devices for real-time monitoring of drug release kinetics and tumor microenvironment changes represents an innovative approach enabling adaptive dosing and personalized treatment optimization [1], while MnO_2_–MgAl nanohybrids have been developed for real-time detection of hydrogen peroxide from live cancer cells [130].

Precise control over structural characteristics including morphology and layer thickness will be crucial for future applications, as 2D ultrathin nanosheets with large surface areas enable higher drug loading and enhanced tumor penetration [131], while plate-like or rod-shaped morphologies show superior cellular internalization and controlled thickness determines biodegradation rates for fine-tuning therapeutic release [65]. The emerging field of CRISPR-Cas9 delivery using LDHs offers significant potential for cancer gene therapy, leveraging their biocompatibility, high nucleic acid loading capacity, and pH-sensitive release to protect and deliver gene-editing components while minimizing off-target effects [132,133], with recent studies demonstrating effective knockout of oncogenes like KRAS and restoration of tumor suppressors like TP53 [39,134], though challenges remain in endosomal release, editing efficiency in heterogeneous tumors, and immune activation [135,136]. The integration of artificial intelligence in LDH design represents a paradigm shift toward personalized cancer therapy, as machine learning algorithms can process complex biological datasets to predict optimal drug combinations and tailor formulations to individual patient profiles [137], enabling precise adjustment of particle size, surface charge, and release kinetics while facilitating rapid screening of synergistic drug pairs and creation of co-delivery systems that address tumor heterogeneity more effectively [116]. Collectively, these advances position LDH nanoplatforms as versatile, customizable systems for precision oncology, offering integrated therapeutic, diagnostic, and monitoring capabilities that promise to transform cancer treatment from symptom management to curative interventions through their structural tunability, multimodal therapy capacity, and ability to integrate advanced technologies for next-generation personalized medicine.

## 7 Conclusions

LDHs have evolved from conventional drug carriers into next-generation multifunctional nanoplatforms with transformative potential in cancer therapy. Their tunable physicochemical features such as ion-exchangeable lamellar structures, structural adaptability, high loading capacity, and intrinsic pH responsiveness allow for precise delivery of a broad spectrum of therapeutics while minimizing systemic toxicity. A defining advantage of LDHs is their modular design which enables the co-delivery of multiple therapeutic and diagnostic agents within a single nanocarrier. This versatility underpins the development of synergistic combination therapies including PTT, PDT, CDT, immunotherapy, and gene therapy, each targeting distinct tumor weaknesses. Moreover, LDHs have shown promise as carriers for gene-editing systems such as CRISPR-Cas9, offering safe, pH-triggered nucleic acid delivery with lower immunogenic risk compared to viral vectors.

What distinguishes LDHs from other 2D nanomaterials like graphene oxide, black phosphorus, or MXenes is their unique combination of stability, biocompatibility, and functionality. Unlike these systems, LDHs possess a positively charged, ion-exchangeable framework that enables efficient intercalation of anionic drugs and genetic cargo. Their structural tunability allows the integration of therapeutic ions for multimodal therapies, while their superior chemical stability and biodegradation into biocompatible ions (e.g., Mg^2+^, Ca^2+^) address safety concerns that often limit other platforms. These attributes make LDHs especially suitable for designing theranostic systems that unite targeted therapy, real-time imaging, and tumor-specific responsiveness. Looking ahead, key advances will come from engineering LDHs with precise control over morphology, layer thickness, and defect density to further optimize biodistribution, cellular uptake, and therapeutic payload release. The convergence of LDH design with AI-assisted modeling, wearable biosensors, and patient-tailored medicine heralds a future of intelligent, adaptive cancer therapies. However, challenges such as large-scale reproducibility, regulatory approval, and comprehensive understanding of long-term safety must be addressed to fully realize their clinical potential. Overall, LDHs stand poised as pivotal materials for the next generation of precision cancer therapy, combining structural versatility with multimodal functionality to move beyond symptom management toward durable, patient-specific cures.

## CRediT authorship contribution statement

**Nazila Biglari:** Writing – original draft, Formal analysis, Conceptualization. **Matineh Ghomi:** Investigation, Data curation. **Ehsan Nazarzadeh Zare:** Writing – review & editing, Conceptualization. **Elham Mahmoudi:** Writing – original draft. **Jianliang Shen:** Writing – review & editing. **Pooyan Makvandi:** Writing – review & editing.

## Funding sources

This study was not supported by any funding.

## Declaration of competing interests

Pooyan Makvandi and Jianliang Shen are editorial board members for Bioactive Materials and was not involved in the editorial review or the decision to publish this article. All authors declare that there are no competing interests.

## Data Availability

No data were generated.
